# Identification of three classes of acute respiratory distress syndrome using latent class analysis

**DOI:** 10.7717/peerj.4592

**Published:** 2018-03-30

**Authors:** Zhongheng Zhang

**Affiliations:** Department of Emergency Medicine, Sir Run Run Shaw Hospital, Zhejiang University School of Medicine, Hangzhou, China

**Keywords:** Latent class analysis, Acute respiratory distress syndrome, Fluid balance, Mortality

## Abstract

Acute respiratory distress syndrome (ARDS) is a highly heterogeneous syndrome that can exhibit significant differences in the underlying causes, leading to different responses to treatment. It is required to identify subtypes of ARDS to guideline clinical treatment and trial design. The study aimed to identify subtypes of ARDS using latent class analysis (LCA). The study was a secondary analysis of the EDEN study, which was a randomized, controlled, multicenter trial conducted from January 2, 2008 to April 12, 2011. The primary study endpoint was death through 90-day follow up. LCA was performed incorporating variables on day 0 before randomization. The number of classes was chosen by a bootstrapped likelihood ratio test, Bayesian information criterion and the number of patients in each class. A total of 943 patients were enrolled in the study, including 219 (23.2%) non-survivors and 724 (76.8%) survivors. The LCA identified three classes of ARDS. Class 1 (hemodynamically unstable type) had significantly higher mortality rate (*p* = 0.003) than class 2 (intermediate type) and 3 (stable type) through 90 days follow up. There was significant interaction between cumulative fluid balance and the class (*p* = 0.02). While more fluid balance was beneficial for class 1, it was harmful for class 2 and 3. In conclusion, the study identified three classes of ARDS, which showed different clinical presentations, responses to fluid therapy and prognosis. The classification system used simple clinical variables and could help to design ARDS trials in the future.

## Background

Acute respiratory distress syndrome (ARDS) is a clinical syndrome manifested by acute onset of respiratory failure requiring medical intervention. Clinical outcomes may be greatly improved if ARDS is promptly identified at an early stage and treated properly. Despite strenuous efforts being made to improve the clinical outcomes of ARDS (Steinberg et al., 2006; Mansur et al., 2015; Zhang, Chen & Ni, 2015; Kacmarek et al., 2016; Artigas et al., 2017), the mortality rate remains unacceptably high (Frenzel et al., 2011; Dalhoff et al., 2012; Bellani et al., 2016; Wang et al., 2016). The recent LUNG SAFE study reports a hospital mortality of 40%, with an increase across the severity in the Berlin definition (Bellani et al., 2016). There are only a few interventions being proven to be effective in improving survival outcome which includes protective mechanical ventilation, conservative fluid management, neuromuscular blockade and prone positioning (Acute Respiratory Distress Syndrome Network et al., 2000; Wang et al., 2016; Murray et al., 2016; Matthay, McAuley & Ware, 2017). The challenge in the treatment of ARDS is probably attributable to the heterogeneity of the population. ARDS is classified as a syndrome simply because these patients share some common features in clinical presentations such as the acute onset, diffuse bilateral infiltrates in chest X-ray and low oxygenation as reflected by the PaO_2_/FiO_2_ (P/F) ratio (ARDS Definition Task Force et al., 2012). As a matter of fact, ARDS is a heterogeneous clinical syndrome exhibiting quite different clinical courses and responses to treatment, depending on the underlying causes, disease stage and comorbidities (Meyer & Christie, 2013; Shaver & Bastarache, 2014; Luo et al., 2017). Thus, it is important to find subclass of ARDS patients who are more likely to benefit from treatments, presumably because the subclass has a different underlying pathophysiology (Russell et al., 2017).

Latent class analysis (LCA) can help to find classes or subtypes of cases in multivariate data. It has been widely used in economics, business and psychology (Krauss et al., 2017; Nouwens et al., 2017). However, LCA is not widely understood in medicine, and there are often inadequate feature variables to characterize clinically meaningful latent classes. A variety of clinical and laboratory variables were collected in clinical trials involving ARDS patients. These data provided a good opportunity to establish a latent class model to identify subtypes of ARDS that can have different treatment responses. Previous studies have successfully classified ARDS into subphenotypes (Calfee et al., 2014; Famous et al., 2017). However, these studies employed inflammatory biomarkers such as interleukins and tumor necrosis factor which were not routinely available in routine clinical practice and the classification system might not be widely applicable. In this study, the author aimed to identify subtypes of ARDS by using simple clinical and laboratory variables. Furthermore, the mortality outcome was compared between subtypes. The response to fluid balance was also compared between ARDS subtypes.

## Methods

### Study population

The study was a secondary analysis of the early versus delayed enteral feeding to treat people with acute lung injury or acute respiratory distress syndrome (EDEN) study, which was a randomized, controlled, multicenter trial conducted from January 2, 2008 to April 12, 2011 (National Heart, Lung, and Blood Institute Acute Respiratory Distress Syndrome (ARDS) Clinical Trials Network et al., 2012). The study randomized 1,000 patients within 48 h of developing ARDS requiring mechanical ventilation in approximately equal numbers to receive either trophic or full enteral feeding for the first six days. The effectiveness of both interventions was comparable with regard to clinical outcomes such as 60-day mortality, ventilator-free days and infectious complications. The secondary analysis utilized de-identified patient information and was approved by the institutional review board of Sir Run Run Shaw Hospital (20170313-2).

### Definition of some variables

The study recorded three types of patient status at 90 days which were home with unassisted breathing, dead prior to home with unassisted breathing and last known status (neither dead nor home with unassisted breathing). For the purpose of de-identification, the date of these status was transformed to the number of days by subtracting the date of randomization. Death was considered as the event of interest.

Variables recorded on day 0 (before randomization) including age, heart rate, temperature, systolic blood pressure, diastolic blood pressure, sodium (plasma), bicarbonate (plasma), potassium (plasma), albumin (serum) and P/F ratio. If there were multiple records, the one closest to 8 o’clock was used. Fluid intakes and outputs were obtained for the first 24 h after randomization. Fluid balance for a single day was calculated as the difference between fluid intake and output.

### Statistical analysis

Continuous variables were examined for their normality. Normal data were expressed as mean and standard deviation, and skewed data were expressed as median and interquartile range (Zhang, 2016a). Mann–Whitney *U* tests or *t*-tests were used for two group comparisons as appropriate. Variables normally distributed within latent classes were compared using analysis of variance, and non-normal data were compared using the Kruskal–Wallis test. Categorical variables were expressed as the number and proportions. Proportions were compared using Chi-square test or Fisher’s exact test (e.g., the former was used as long as 80% or more of cells had expected cell counts greater than five and all observed cell counts were at least one). Comparisons between latent classes were performed using the CBCgrps package in R (Zhang et al., 2017).

Latent class analysis is a subset of structural equation modeling where observed variables are used to identify unobserved or latent classes (Zhang, 2017). The number of classes was determined by using the Bayesian information criterion (BIC), a parametric bootstrapped likelihood ratio test, relative entropy and the number of individuals within each class (Nylund, Asparouhov & Muthén, 2007). The expectation-maximization algorithm was used to obtain the maximum likelihood estimates for the parameters. The distributions of observed variables were displayed across identified classes of ARDS.

A multivariable logistic regression model was built to examine the adjusted differences in 90-day mortality between ARDS classes (Zhang, 2016b). The interaction between class membership and randomization group were explored in the logistic regression model. To investigate the influence of ARDS classes on the effect of fluid balance on mortality risk, another logistic regression model was established by including the interaction between class and fluid balance on day 1.

All statistical analyses were performed using R (version 3.3.2) (R Core Team, 2016). Two-tailed *p* values less than 0.05 were considered statistically significant.

## Results

A total of 943 patients were included in the study, including 219 (23.2%) non-survivors at 90 days and 724 (76.8%) survivors. Non-survivors were significantly older (60 (47, 72) vs. 51 (41, 60) years, *p* = 0.001), had higher baseline heart rate (98 (84, 110) vs. 94 (80, 108) per minute; *p* = 0.001), and potassium levels (4.0 (3.6, 4.5) vs. 3.9(3.6, 4.3) mmol/l; *p* = 0.07) than non-survivors. Non-survivors had significantly lower values of mean blood pressure (74 (67, 80) vs. 76 (69, 84) mmHg; *p* < 0.001) and P/F ratio (130 (92, 178) vs. 148 (108, 203); *p* = 0.002) than survivors (Table 1).

**Table 1:** Baseline characteristics between survivors and non-survivors.

Variables	Total (*n* = 943)	Survivors (*n* = 724)	Non-survivors (*n* = 219)	*p*
P/F ratio	144 (105, 200)	148 (108, 203)	130 (92, 178)	0.002
Heart rate (per minute, IQR)	94 (81, 108)	94 (80, 108)	98 (84, 110)	0.081
Mean blood pressure (mmHg)	76 (69, 84)	76 (69, 84)	74 (67, 80)	0.001
Sodium (mmol/l)	138 (135, 142)	138 (135, 142)	139 (135, 142)	0.859
Albumin (mg/dl)	2.2 (1.8, 2.7)	2.3 (1.9, 2.7)	2.1 (1.8, 2.7)	0.010
Potassium (mmol/l)	3.9 (3.6, 4.4)	3.9 (3.6, 4.3)	4.0 (3.6, 4.5)	0.070
Bicarbonate (mmol/l)	22 (19, 26)	22.5 (20, 26)	22 (18, 25)	0.032
Age (years, IQR)	52 (42, 63)	51 (41, 60)	60 (46.5, 72)	0.001

**Note:**

*p* Values were computed using Mann–Whitney *U* tests.

### Latent class analysis

**Figure 1:**
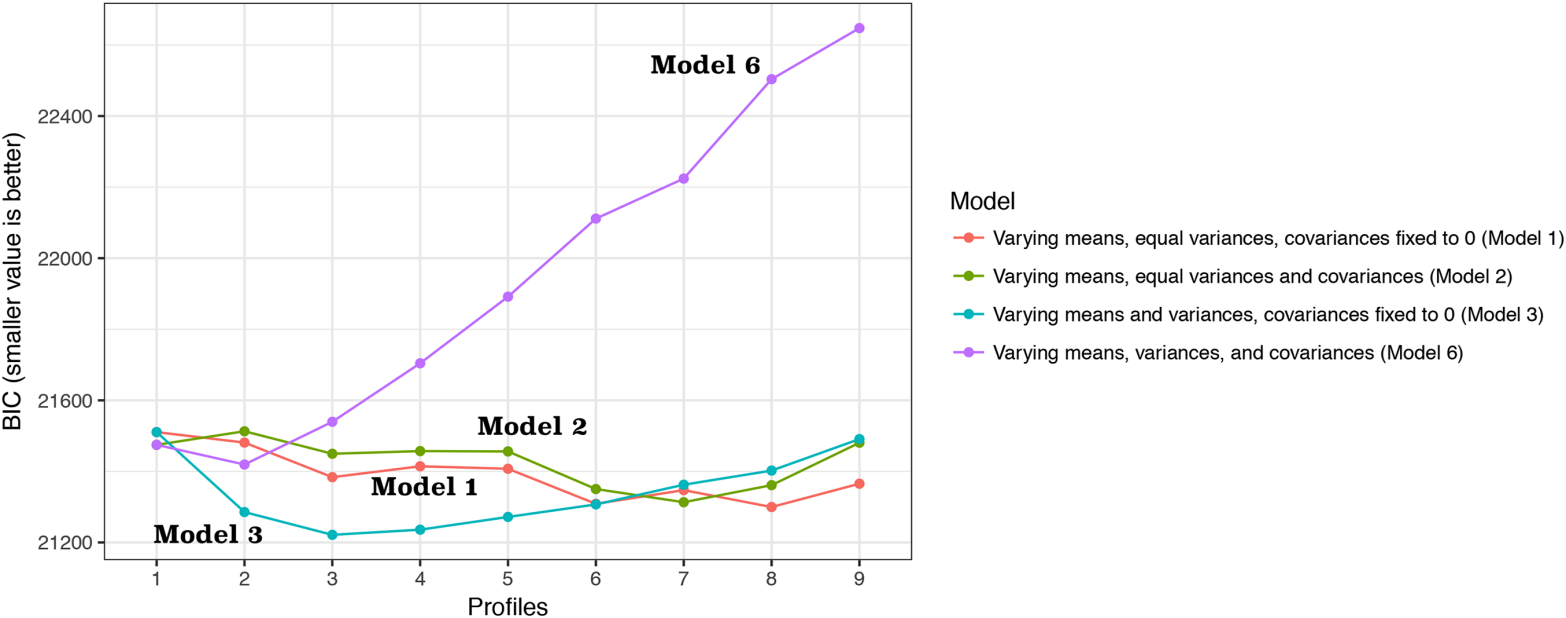
Bayesian information criterion (BIC) for choosing the number of classes. Four models with different assumptions were employed for estimating BIC values. The three-class model showed the lowest BIC values in three models (1, 2 and 3). Model 6 with varying means, variances and covariances showed the lowest BIC value for two-class model.

**Figure 2:**
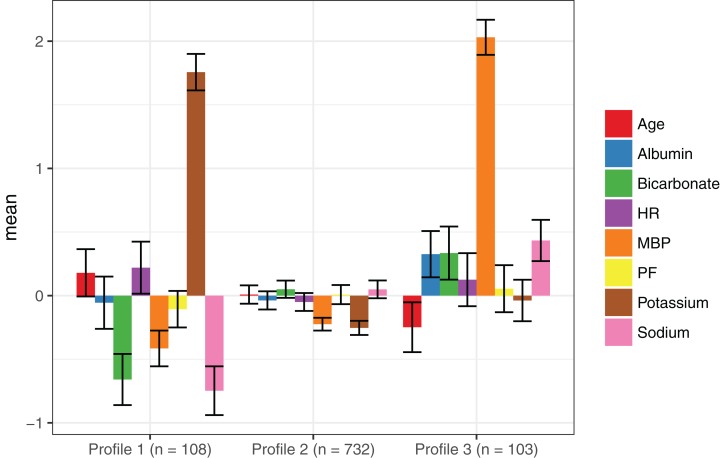
Latent class profile for the three-class model. Class 1 was characterized by older age, lower mean blood pressure, higher heart rate and lower bicarbonate. Class 3 was characterized by younger age, lower heart rate and higher blood pressure. Class 2 is the intermediate type between class 1 and 3. Abbreviations: PF, P/F ratio; HR, baseline heart rate; MBP, mean blood pressure.

The BIC results are shown in Fig. 1. The three-class model showed the lowest BIC values in three models (1, 2 and 3). Model 6 with varying means, variances and covariances showed the lowest BIC value for two-class model. The three-class model had the highest entropy value (0.88). The number of patients in each class was sizable for the three-class model (108, 732 and 103 for class 1, 2 and 3, respectively). The bootstrapped likelihood ratio test also showed that the three-class model was significantly better than two-class model but not worse than four-class model (Table 2). As a result, the three-class model was chosen as the best fit model. Class 1 was characterized by older age, lower mean blood pressure, higher heart rate and lower bicarbonate (Table 3). Class 3 was characterized by younger age, lower heart rate and higher blood pressure. Class 2 was an intermediate type between class 1 and 3. The numbers of individuals were 108, 732 and 103 in class 1, 2 and 3, respectively (Fig. 2). Thus, class 1 could be named the hemodynamically unstable type; class 2 was named the intermediate type and class 3 was the stable type.

**Table 2:** Criteria to choose the best number of classes.

Number of classes	BIC	Entropy	Number of individuals per class						Bootstrapped likelihood ratio test
			1	2	3	4	5	6	*p* Value
2	21481.114	0.78	374	569					0.001
3	21383.724	0.88	108	732	103				0.001
4	21414.326	0.72	228	499	107	109			0.078
5	21407.395	0.75	175	562	86	19	101		0.067
6	21308.773	0.76	179	552	84	19	103	6	0.079

**Notes:**

*p* Values were comparing with model with one fewer class.

BIC, Bayesian information criterion.

**Table 3:** Differences of feature variables among the three latent classes.

Variables	Class 1	Class 2	Class 3	*p* Value
P/F ratio	140 (107, 193)	145 (105, 200)	155 (109, 203)	0.621
Heart rate (per minute, IQR)	98 (83, 116)	94 (80, 106)	97 (80, 112)	0.040
Mean blood pressure (mmHg)	71 (65, 78)	74 (68, 81)	102 (98, 107)	0.001
Sodium (mmol/l)	135 (131, 139)	139 (136, 142)	141 (138, 143)	0.001
Albumin (mg/dl)	2.2 (1.7, 2.7)	2.2 (1.8, 2.7)	2.4 (2.1, 3.0)	0.001
Potassium (mmol/l)	5.0 (4.8, 5.4)	3.8 (3.5, 4.2)	4 (3.6, 4.4)	0.001
Bicarbonate (mmol/l)	19 (15, 22)	23 (20, 26)	24 (20, 27)	0.001
Age (years, IQR)	55 (45, 68)	52 (42, 63)	51 (37, 58)	0.018
Mortality	36 (0.33)	169 (0.23)	14 (0.14)	0.003

**Notes:**

Continuous variables were expressed as median and interquartile range, and categorical data were expressed as the number and proportions. *p* Values were computed using the Kruskal–Wallis test.

### Clinical characteristics of the three classes

**Figure 3:**
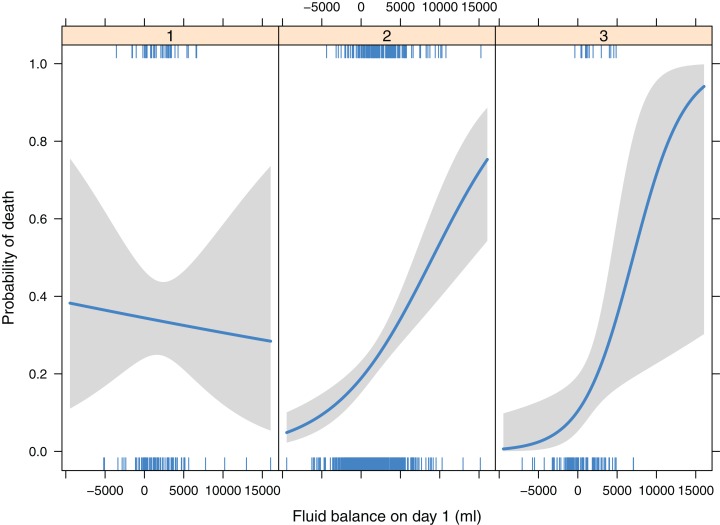
Interactions between ARDS class membership and fluid balance on day 1, by adjusting for randomization group.

Logistic regression showed there was no significant interaction between randomization group and class membership (*p* = 0.553), and this interaction term was removed from the model. As compared with class 3, class 1 (OR: 2.76; 95% CI [1.38–5.82]) was associated with higher risk of death. Class 1 was associated with higher risk of death than class 2 (OR: 1.72; 95% CI [1.09–2.68]). Class 2 and 3 had similar mortality outcome (OR: 1.60; 95% CI [0.90–3.09]; *p* = 0.132). The randomization group had no independent effect on mortality outcome (*p* = 0.557). There was significant interaction between class membership and fluid balance on day 1 (*p* = 0.02). While more positive fluid intake was associated with lower risk of death in class 1, more negative fluid balance was associated with lower risk of death in class 2 and 3 (Table 4; Fig. 3).

**Table 4:** Interaction between fluid balance and latent class in multivariable model with 90-day survival outcome as the response variable.

Variables	Odds ratio	Lower limit of 95% CI	Upper limit of 95% CI	*p* Value
Class 1 vs. 3	2.763	1.377	5.818	0.005
Class 2 vs. 3	1.604	0.895	3.091	0.132
Class 1 vs. 2	1.723	1.093	2.675	0.017
Fluid balance on day 1 with each 2 l increase for class 1	0.966	0.726	1.260	0.801
Fluid balance on day 1 with each 2 l increase for class 2	1.378	1.211	1.574	0.001
Fluid balance on day 1 with each 2 l increase for class 3	1.849	1.146	3.132	0.015
Randomization group (intervention vs. control)	0.911	0.667	1.243	0.557

**Notes:**

*p* Values were computed using Wald statistic.

CI, confidence interval.

## Discussion

The study identified three classes of ARDS that had different 90-day mortality risk (e.g., class 1 had significantly higher mortality rate than class 2 and 3). Class 1 was a hemodynamically unstable type characterized by lower mean blood pressure and higher heart rate. Class 2 was an intermediate type that the SIRS response appears less severe than class 1. Class 3 was a group with stable vital signs. Multivariable regression model showed that while more fluid balance is potentially beneficial for class 1, more fluid balance was associated with increased risk of death for classes 2 and 3. The identification of these subtypes of ARDS may help to triage ARDS patients that respond differently to fluid therapy, and to design future clinical trials by using simple clinical variables.

It has long been noticed that ARDS is not a homogenous disease (Rezoagli, Fumagalli & Bellani, 2017), but it encompasses a highly heterogeneous patient population who have remarkable differences in genetic background, clinical characteristics, treatment responses and mortality outcomes (Meyer & Christie, 2013; Shaver & Bastarache, 2014; Matthay, McAuley & Ware, 2017). ARDS is clinically classified as a syndrome simply because it describes a collection of commonly encountered symptoms in critically ill patients, which typically involves acute onset, bilateral lung infiltrates, low oxygenation and requirement of respiratory support. Since ARDS is a heterogeneous clinical syndrome, many efforts have been made to categorize it into subtypes or classes. Brown and colleagues classified ARDS patients into four subtypes according to their six-month health status (Brown et al., 2017). However, this classification system required data over a six-month period and cannot be used at the beginning of disease onset. The strength of the present study was that clinical variables collected at the early stage of ARDS onset were employed, which can help clinicians to identify subtypes of ARDS that have different clinical outcomes and treatment responses. In another study, the authors employed inflammatory biomarkers to classify ARDS into two subphenotypes and the results showed that these subphenotypes had differences in clinical outcomes and responses to PEEP strategy (Calfee et al., 2014). Type 2 was characterized by hyperinflammatory responses, corresponding to the class 1 in our study. They further investigated the difference in the effect of liberal versus conservative fluid treatment strategy on clinical outcomes between the two subphenotypes. Fluid management strategy had significantly different effects on 90-day mortality in the two subphenotypes (*p* = 0.0039 for interaction). While conservative strategy resulted in higher mortality than liberal strategy in subphenotype 1 (26% vs. 18%), mortality in subphenotype 2 was lower with fluid-conservative strategy than the liberal strategy (40% vs. 50%). In this study, more positive fluid balance appeared to be associated with reduced risk of death in class 1, while the effect was opposite in class 2 and 3. The adverse effect of positive fluid balance was prominent in class 3 (corresponding to the hypo-inflammatory subphenotype). It is reasonable that ARDS patients without shock can benefit from conservative fluid strategy to reduce pulmonary edema. On the contrary, for patients with unstable hemodynamics, maintaining tissue perfusion by liberal fluid strategy is the priority. However, the biomarkers in Calfee’s study are not available in routine clinical practice, inhibiting its widespread use. In contrast, the present study employed simple variables that were readily available in all clinical institutions, making external validation of the classification system feasible. Famous and colleagues further showed that a parsimonious model involving only three variables interleukin-8, bicarbonate and tumor necrosis factor receptor-1 could accurately identify the two-class model. However, these biomarkers except for bicarbonate are still not routinely tested in real clinical practice, especially in some low-income countries.

Several limitations need to be acknowledged in the study. The study was a secondary analysis of a clinical trial, which was subject to inherent limitations of the post hoc analysis. Sample size and statistical power were not designed for the LCA statistical model. Fluid strategy was not randomized in the original study, raising the problem of selection bias, e.g., more fluid was given to class 1 patients. In this case, class membership is a confounder for the relationship between fluid balance and mortality outcome. In the present study, any such confounding effect was adjusted for by the use of multivariable regression model. Another limitation of the study is that LCA results are not readily transferable to a score or some other algorithm to help triage ARDS patients. The next step may be to build a multinomial logistic regression model by regressing latent class on candidate predictors to obtain the weight (regression coefficient) for each of the predictors. In this way, a mathematical equation or nomogram can be built to predict the probability of belonging to a subclass for a given patient with known predictors (Zhang & Kattan, 2017).

## Conclusion

In conclusion, this study identified three classes of ARDS, which showed different clinical outcomes and treatment responses to fluid therapy. The classification system used simple clinical variables and could help to design ARDS trials in the future.

## Additional Information and Declarations

### Clinical Trial Registration

The following information was supplied regarding Clinical Trial registration: Trial registration: NCT00609180, registered February 6, 2008.

### Data Availability

The following information was supplied regarding data availability: All data are available at: https://biolincc.nhlbi.nih.gov/studies/eden/?q=EDEN.
